# Incidence of Hip Fractures among Patients with Chronic Otitis Media: The Real-World Data

**DOI:** 10.3390/medicina58081138

**Published:** 2022-08-22

**Authors:** Pei-Shao Liao, Ching-Chih Chiu, Yi-Hsiu Fu, Chia-Chun Hsia, Yu-Cih Yang, Kun-Feng Lee, Shang-Lin Hsieh, Shu-Jui Kuo

**Affiliations:** 1 Department of Otolaryngology Head and Neck Surgery, China Medical University Hospital, Taichung 404327, Taiwan; 2 Department of Education, China Medical University Hospital, Taichung 404327, Taiwan; 3 Department of Education, Taichung Veterans General Hospital, Taichung 407219, Taiwan; 4 Management Office for Health Data, China Medical University Hospital, Taichung 404327, Taiwan; 5 School of Medicine, China Medical University, Taichung 404328, Taiwan; 6 Department of Orthopedic Surgery, China Medical University Hospital, Taichung 404327, Taiwan

**Keywords:** chronic otitis media, hearing loss, hip fracture, National Health Insurance Research Database, osteoporosis

## Abstract

Chronic otitis media (COM) has been considered as a localized disease, and its systemic impact is poorly understood. Whether COM-induced inflammation could be associated with systemic bone loss and hip fracture is unknown at present. Our study tried to determine the risk of hip fracture among COM patients. We selected the comparison individuals without the COM coding and paired the controls with COM patients by gender, age, and comorbidities (including osteoporosis) by about a one-to-two ratio. Our study showed that the incidence of hip fracture was 4.48 and 3.92 per 1000 person-years for comparison and COM cohorts respectively. The cumulative incidence of hip fracture is higher in the COM cohort (*p* < 0.001). After adjustment for gender, age, and comorbidities, the COM patients had a 1.11-fold (aHR = 1.11; 95% CI = 1.05–1.17) risk of hip fracture than the control subjects. Among COM patients, a history of hearing loss is associated with higher (aHR = 1.21; 95% CI = 1.20–1.42) fracture risk. Our study showed that COM patients, especially those with hearing loss, are susceptible to a higher risk for hip fracture.

## 1. Introduction

Chronic otitis media (COM) is manifested by inflammation of the middle ear due to infectious or non-infections processes, resulting in pathologic changes in the tympanic membrane [1]. The pathologic changes of the tympanic membrane secondary to COM include atelectasis, perforation, retraction, tympanosclerosis, and cholesteatoma [1,2]. COM is also an important etiology of acquired hearing loss and is the main disease in the field of otolaryngology [1]. COM can lead to potentially deadly complications such as mastoiditis, petrositis, labyrinthitis, meningitis, brain abscess, and thrombophlebitis [1]. Despite the potentially fatal nature of the complications mentioned above, these complications are local in nature and the systemic effects of COM are unknown currently.

Bone loss is a recognized consequence of systemic inflammatory processes, which could be explained by the perspective of evolution, disturbed energy expenditure and storage, and immunologic as well as neuroendocrine factors [3]. There is also a genetic linkage between auditory tract disease and bone loss. For example, FBXO11 is associated with the susceptibility to otitis media and affects osteoblastogenesis [4,5,6]. The correlations between inflammation, shared genetic factors, and the impact of COM on bone metabolism have not been rigorously determined.

The most devastating complication secondary to bone loss is hip fracture [7]. Hip fractures are accountable for 25% of geriatric fractures mandating hospitalization with high morbidity and mortality [8]. There is no available evidence demonstrating whether COM-induced inflammation could be associated with systemic bone loss and hip fracture. In our study, we want to investigate the incidence of hip fracture among subjects with COM. We thus conducted a nationwide population-based study, attempting to unravel the association between COM and hip fracture. We hypothesize that COM patients might be predisposed to a higher risk of hip fracture.

## 2. Materials and Methods

The Longitudinal Health Insurance Database (LHID) of the National Health Insurance Research Database (NHIRD) was employed for our study under the approval of the Institutional Review Board of China Medical University, Taichung, Taiwan (CMUH104-REC2–115). The LHID is composed of the registry data for the beneficiaries, inpatient and outpatient files, and the registry for drug prescriptions as well as medical services. The history of medical comorbidities of the insured individuals was retrieved from the inpatient and outpatient files. The International Classification of Diseases, 9th Revision, Clinical Modification (ICD-9-CM) system was applied as the disease-coding system in NHIRD. The original identification numbers were eliminated to safeguard the privacy of the insured citizens, and a scrambled number was offered to correlate the claim data to each individual before releasing the data for research purposes.

A population-based cohort study was established to determine the incidence of hip fracture (ICD-9-CM code 820.x) among the subjects with COM. All of the patients who were coded with COM (ICD-9-CM code 382.1–382.9) first in their lifetime from 1 January 2000 to 31 December 2013, were assessed. The date when the diagnosis of COM was initially coded was defined as the index date. The comparison individuals without the diagnostic coding of COM were chosen from the LHID and paired with COM patients by gender, age (exact year), and the coding of osteoporosis (ICD-9-CM 733.0 and 733.1) at the index date by about a one-to-two ratio. The comparison subjects were recruited on the same date as the paired COM patients. All of the enrolled individuals were followed until the withdrawal from the insurance, the advent of hip fracture, or until 31 December 2013. The comorbidities analyzed in our study included hypertension (ICD-9-CM code 401–405), diabetes (ICD-9-CM code 250), epilepsy (ICD-9-CM code 345, A225), ischemic heart disease (ICD-9-CM code 410–414), chronic obstructive pulmonary disease (COPD, ICD-9-CM code 491, 492, 493 and 496), stroke (ICD-9-CM code 430–438), liver cirrhosis (ICD-9-CM code 571 and 572), osteoporosis (ICD-9-CM code 733) and end-stage renal disease (ICD-9-CM code 585.6). The impact of hearing loss (ICD-9-CM code 389), vertigo (ICD-9-CM code 386), and tinnitus (ICD-9-CM code 388.3) on the occurrence of hip fractures among COM patients were also analyzed.

The incidence rate of hip fracture was expressed as events per 1,000 person-years. The chi-square test for categorical variables and the two-sample *t*-test for continuous variables were applied for between-group comparisons. The Kaplan–Meier method was utilized to plot the cumulative incidence of hip fractures, and the log-rank test was employed to determine the significance of between-group differences [9,10]. To demonstrate the risk of hip fracture for the COM and control cohorts, the crude and adjusted hazard ratios (cHRs and aHRs) and 95% confidence intervals (CIs) were calculated by employing the single- and multi-variable Cox proportional hazard models [11]. The SAS 9.4 software (SAS Institute, Cary, NC, USA) was applied for data analysis and the R software (R Foundation for Statistical Computing, Vienna, Austria) was employed to plot the incidence curves.

## 3. Results

**Figure 1:**
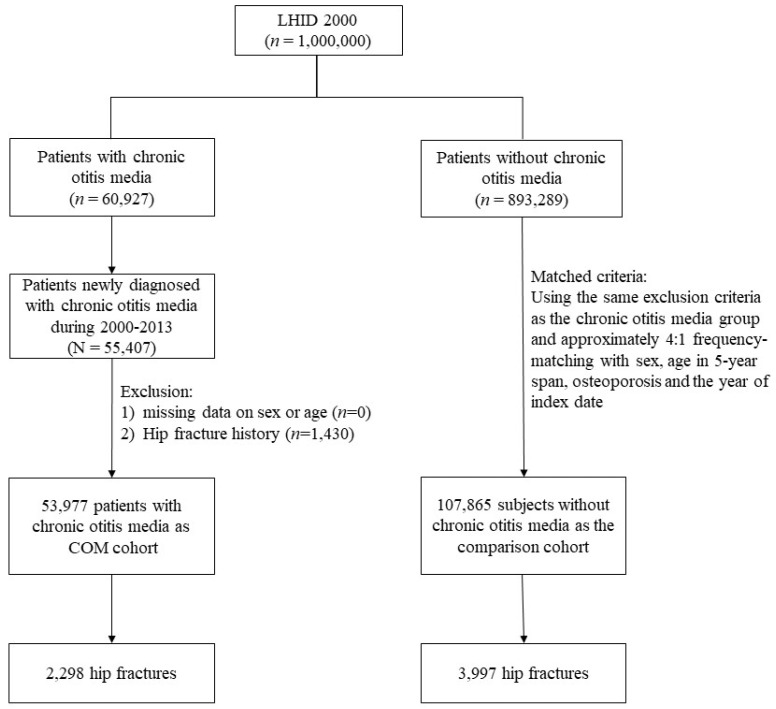
The flow diagram of the recruitment process.

There were 53,997 patients in the COM cohort and 107,865 individuals in the comparison cohort. The distribution of gender, age, and the percentage with the initial diagnosis of osteoporosis were comparable between the two cohorts. The mean age of COM and comparison cohorts were 27.8 ± 86.0 and 28.1 ± 84.4 years, respectively (Table 1). The prevalence of hypertension, diabetes, epilepsy, ischemic heart disease, chronic obstructive pulmonary disease, stroke, and liver cirrhosis were all higher in the COM cohort (all *p* < 0.001). The follow-up duration was 9.49 ±3.7 years for the COM cohort and 9.43 ± 3.7 years for the comparison cohort. The flow diagram for the subject recruitment process was shown in Figure 1.

**Figure 2:**
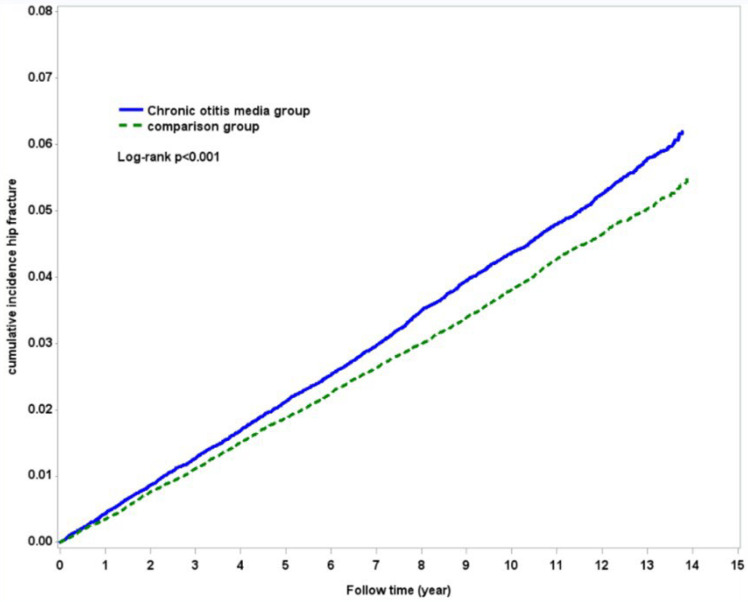
The incidence of hip fracture among COM and comparison cohorts. The dashed line indicates the comparison cohort, and the solid line indicates the COM cohort.

There were 2298 hip fractures in the COM cohort with an incidence density of 4.48 per 1000 person-years. As for the comparison cohort, 3997 individuals suffered from hip fractures with an incidence density of 3.92 per 1000 person-years (Table 2). The cumulative incidence of hip fracture was higher in the COM cohort than in the comparison cohort (Figure 2, *p* < 0.001 for log-rank test). After adjustment for gender, age, and all of the enlisted comorbidities, the subjects in the COM cohort had a 1.11-fold risk of hip fracture than the individuals in the comparison cohort (aHR = 1.11; 95% CI = 1.05–1.17). Age, gender, hypertension, diabetes, epilepsy, chronic obstructive pulmonary disease, stroke, and osteoporosis were also associated with a higher incidence of hip fracture in our model.

Table 3 showed that the impact of COM and on the risk of hip fracture was more pronounced among subjects with female gender (aHR = 1.17; 95% CI = 1.09–1.26), hypertension (aHR = 1.14; 95% CI = 1.05–1.24), diabetes (aHR = 1.19; 95% CI = 1.07–1.32), ischemic heart disease (aHR = 1.15; 95% CI = 1.00–1.33), and chronic obstructive pulmonary disease (aHR = 1.14; 95% CI = 1.02–1.27).

Among the COM patients, a history of hearing loss is associated with a higher (aHR = 1.21; 95% CI = 1.20–1.42) risk of hip fracture (Table 4).

## 4. Discussion

COM has been considered as a localized disease, and its systemic impact is poorly understood. In our study, we showed that the COM patients were subject to 1.11-fold the risk of suffering from hip fracture than the comparison individuals (95% CI = 1.05–1.17). The impact of COM was more prominent among the subjects of female gender, hypertension, diabetes, ischemic heart disease, and chronic obstructive pulmonary disease. Among the COM patients, the history of hearing loss furtherly increased the risk of hip fracture. These results indicate that COM is not merely a “localized” disease.

The pathophysiologic linkage between COM and osteoporotic hip fracture is not clear at present. However, the inflammation and genetic factor could potentially be the pathophysiologic linkage between COM and hip fracture.

As for inflammation, the “three-pillar theory” has been proposed to account for the bone loss under inflammation, including evolution, disturbed energy expenditure and storage, and immunologic as well as endocrine factors [3]. Inflammation has been shown to be involved in the pathogenesis of both auditory tract disease and systemic bone loss. Wang et al. have shown that the osteoporosis cohort displayed a 1.32-fold risk to suffer from acquired cholesteatoma compared with the control cohort (aHR = 1.32; 95% CI = 1.11–1.57), thus suggesting that otolaryngologists should examine the middle ear of osteoporotic patients [12]. Additionally, cholesteatoma is characterized by bone erosion in the middle ear, and increased expression of receptor activator for nuclear factor-κB ligand (RANKL) and osteoprotegerin (OPG) is found in tissues of cholesteatoma [13,14,15]. These results nicely demonstrated the correlation between bone loss and the inflammation of the auditory tract. In our study, we demonstrated that the COM patients had 1.11 times the risk of suffering from hip fracture when compared with the paired comparison individuals. Our study supplemented the previous publications in bridging the association between the inflammation of the auditory tract and bone disease.

A shared genetic factor could be the potential nexus between COM and systemic bone loss. Otitis media (recurrent acute otitis media, chronic otitis media with effusion) is largely associated with genetic susceptibility (40–70%) [16]. Mahmood et al. demonstrated the association between COM and the genetic loci of FBXO11 and TGIF1, both of which are involved in the transforming growth factor -beta (TGF-β) signaling via regulating the Smad proteins [17]. Smad proteins are involved in osteoclastogenesis via the RANKL–RANK interplay [18]. FBXO11 can inhibit the transcriptional activity of the p53 gene via promoting its neddylation as a function of Nedd8-ligase and affects its synergistic cooperation with phospho-Smad2 protein on TGF-β signaling [19]. Phospho-Smad2 could enhance the gene expression specific to osteoclast, resulting in inflammatory bone destruction [18]. Furthermore, p53 itself also suppresses osteoblastogenesis and thus results in reduced bone formation [6,20]. As for TGIF1, it has also been shown that TGIF1 knockout mice could develop COM with suppressed Smad expression in middle ear epithelial cells [21]. Vertigo and dizziness are the clinical manifestations of COM, and a previous study revealed that they could increase the risk of falling [22]. The associations between genetic factors, COM, precipitation to falling, and hip fracture warrant further investigations.

We also displayed that the history of hearing loss coincided with a higher risk of hip fracture among the COM patients. Previous studies have shown that osteoporosis was associated with decreased ossicle mass, decreased bone density of the cochlea, and disturbed sound transmission to the cochlea, which end up having a higher incidence of a sensorineural type of hearing loss [23,24]. Yoo et al. analyzed 2588 women and 2273 men aged over 50 years to determine the correlation between senile hearing loss and osteoporosis. The authors demonstrated that individuals with decreased femoral neck bone mineral density could sustain a 1.7-fold risk of suffering from hearing loss (*p* < 0.01) [20]. In our study, the history of hearing loss is associated with a higher (aHR = 1.21; 95% CI = 1.20–1.42) risk of suffering from hip fracture among COM patients. The shown impact of hearing loss on the onset of hip fracture among COM patients in our study supplemented the knowledge concerning the correlation between hearing loss and bone disease.

Our study is not free from limitations. First, the NHIRD did not include all the known risk factors for hip fracture. Some important risk factors, such as vitamin D deficiency and family history, could not be retrieved from the NHIRD. In fact, it is impossible and not necessary to include every known risk factor of hip fracture for analysis. The optimal way to determine the impact of COM on the occurrence of hip fracture was propensity score matching. The absence of propensity score matching would raise the concern that the association between COM and hip fracture might be secondary to the un-recognized medical comorbidities associated with COM. However, excessive matching could skew the generalizability of our findings, so we only paired the coding of osteoporosis in this study and rigorously adjusted all the retrieval comorbidity factors in our regression model instead. Our group has been dedicated to unraveling the association between diseases that can stimulate inflammatory responses and hip fracture. Our future work will continue to unravel more disease entities that could predispose patients to hip fracture. We hope that our work can help in optimizing the risk prediction of, or even prevent, hip fracture.

## 5. Conclusions

✓ The incidence of hip fracture was 4.48 and 3.92 per 1000 person-years for comparison and COM cohorts respectively. After adjustment for gender, age, and comorbidities, the COM patients had a 1.11-fold (aHR = 1.11; 95% CI = 1.05–1.17) risk of hip fracture than the control subjects.
✓ The cumulative incidence of hip fracture is higher in the COM cohort (*p* < 0.001) than in the comparison cohort.
✓ Among the COM patients, a history of hearing loss is associated with higher (aHR = 1.21; 95% CI = 1.20–1.42) fracture risk.

## Figures and Tables

**Table 1:** Demographic profiles for the COM and comparison cohorts.

	COM Cohort		Comparison Cohort		
	*n* = 53,977		*n* = 107,865		*p*-Value *
	*n*	%	*n*	%	
Gender					0.820
Female	27,443	50.8	54,885	50.9	
Male	26,554	49.2	52,980	49.1	
Age					0.999
<20	26,132	48.4	52,135	48.3	
20–44	12,896	23.8	25,792	23.9	
45–64	10,154	18.8	20,308	18.8	
≥65	4815	8.92	9630	8.93	
mean(SD) ^†^	27.84 (86.0)		28.11 (84.4)		<0.001
Comorbidities					
Hypertension	11,988	22.2	21,349	19.7	<0.001
Diabetes	7024	13.0	12,033	11.1	<0.001
Epilepsy	453	0.84	638	0.59	<0.001
Ischemic heart disease	3854	7.14	6120	5.67	<0.001
COPD	10,639	20.3	14,314	13.2	<0.001
Stroke	2392	4.43	3899	3.61	<0.001
Liver cirrhosis	5576	10.3	8724	8.09	<0.001
Osteoporosis	1712	3.17	3422	3.17	0.983
End-stage renal disease	0	0	0	0	-

COM: chronic otitis media; COPD: Chronic obstructive pulmonary disease; SD: standard deviation. ^†^: compared by two-sample *t*-test; *: compared by chi-square test unless marked by ^†^.

**Table 2:** The incidence density of hip fracture stratified by the history of chronic otitis media, gender, age, and comorbidities.

Variable	Hip Fracture			Crude HR (95%CI)	^#^ Adjusted HR (95%CI)
	Hip Fracture	PY	IR		
Chronic otitis media					
No	3997	1,018,072	3.92	1 (reference)	1 (reference)
Yes	2298	512,483	4.48	1.14 (1.08–1.20) ***	1.11 (1.05–1.17) ***
Gender					
Female	3175	777,192	4.08	1 (reference)	1 (reference)
Male	3120	753,363	4.14	1.01 (0.96–1.06)	1.07 (1.02–1.13) **
Age					
<20	2148	858,051	2.50	1 (reference)	1 (reference)
20–44	1113	332,276	3.34	1.37 (1.27–1.47) ***	1.32 (1.23–1.43) ***
45–64	1653	245,380	6.73	2.78 (2.61–2.96) ***	2.21 (2.04–2.39) ***
≥65	1381	94,848	14.5	6.14 (5.74–6.58) ***	4.10 (3.72–4.52) ***
Comorbidities					
Hypertension					
No	3826	1,259,598	3.03	1 (reference)	1 (reference)
Yes	2469	270,957	9.11	3.08 (2.88–3.19) ***	1.21 (1.12–1.30) ***
Diabetes					
No	4793	1,376,069	3.48	1 (reference)	1 (reference)
Yes	1502	154,486	9.72	2.82 (2.66–2.98) ***	1.22 (1.14–1.31) ***
Epilepsy					
No	6240	1,522,127	4.09	1 (reference)	1 (reference)
Yes	55	8428	6.52	1.60 (1.23–2.09) ***	1.44 (1.10–1.88) **
Ischemic heart disease					
No	5538	1,463,746	3.78	1 (reference)	1 (reference)
Yes	757	66,809	11.3	3.06 (2.84–3.31) ***	0.98 (0.89–1.07)
COPD					
No	4994	1,317,988	3.78	1 (reference)	1 (reference)
Yes	1301	212,567	6.12	1.62 (1.53–1.73) ***	1.19 (1.11–1.27) ***
Stroke					
No	5767	1,491,279	3.86	1 (reference)	1 (reference)
Yes	528	39,276	13.4	3.56 (3.25–3.89) ***	1.24 (1.12–1.37) ***
Liver cirrhosis					
No	5502	1,428,513	3.85	1 (reference)	1 (reference)
Yes	793	102,042	7.77	2.05 (1.90–2.21) ***	1.00 (0.92–1.09)
Osteoporosis					
No	5808	1,495,082	3.88	1 (reference)	1 (reference)
Yes	487	35,473	13.7	3.60 (3.28–3.95) ***	1.49 (1.34–1.65) ***
End-stage renal disease					
No	6295	1,530,555	4.11	1 (reference)	1 (reference)
Yes	--	--	--	--	--

COPD: chronic obstructive pulmonary disease; PY: person-years; IR: incidence rate per 1000 PYs; HR: hazard ratio; CI: confidence interval; ** *p* < 0.01, *** *p* < 0.001; ^#^ Adjusted for age, gender, and enlisted comorbidities.

**Table 3:** Differential incidence rate and hazard ratio of hip fracture among the subjects with and without chronic otitis media stratified by gender, age, and comorbidities.

Variable	Chronic Otitis Media						Crude HR (95%CI)	^#^ Adjusted HR (95%CI)
	No			Yes				
	Hip Fracture	PY	IR	Hip Fracture	PY	IR		
Gender								
Female	1980	517,377	3.82	1195	259,815	4.59	1.20 (1.11–1.29) ***	1.17 (1.09–1.26) ***
Male	2017	500,695	4.02	1103	252,668	4.36	1.08 (1.00–1.16) *	1.05 (0.97–1.13)
Age								
<20	1389	570,608	2.43	759	287,443	2.64	1.08 (0.99–1.18)	1.07 (0.98–1.17)
20–44	675	220,884	3.05	438	111,392	3.93	1.28 (1.14–1.45) ***	1.23 (1.09–1.39) ***
45–64	1045	163,603	6.38	608	81,777	7.43	1.16 (1.05–1.28) **	1.12 (1.01–1.24) *
≥65	888	62,977	14.10	493	31,871	15.46	1.09 (0.98–1.22)	1.06 (0.95–1.19)
Comorbidity								
Hypertension								
No	2492	844,254	2.95	1334	415,344	3.21	1.08 (1.01–1.16) *	1.08 (1.01–1.16) *
Yes	1505	173,818	8.65	964	97,139	9.92	1.14 (1.05–1.24) ***	1.14 (1.05–1.24) **
Diabetes								
No	3106	920,361	3.37	1687	455,708	3.70	1.09 (1.03–1.16) **	1.08 (1.02–1.15) **
Yes	891	97,711	9.11	611	56,775	10.7	1.18 (1.06–1.30) **	1.19 (1.07–1.32) ***
Epilepsy								
No	3964	1,013,163	3.91	2276	508,964	4.47	1.14 (1.08–1.20) ***	1.11 (1.05–1.17) ***
Yes	33	4909	6.72	22	3519	6.25	0.93 (0.54–1.60)	1.02 (0.59–1.77)
IHD								
No	3559	977,529	3.64	1979	486,216	4.07	1.11 (1.05–1.18) ***	1.10 (1.04–1.16) ***
Yes	438	40,543	10.8	319	26,267	12.1	1.12 (0.97–1.29)	1.15 (1.00–1.33) *
COPD								
No	3287	900,652	3.64	1707	417,335	4.09	1.12 (1.05–1.18) ***	1.10 (1.04–1.17) ***
Yes	710	117,420	6.04	591	95,148	6.21	1.02 (0.92–1.14)	1.14 (1.02–1.27) *
Stroke								
No	3675	994,043	3.69	2092	497,236	4.20	1.13 (1.07–1.20) ***	1.11 (1.05–1.17) ***
Yes	322	24,029	13.4	206	15,247	13.5	1.00 (0.84–1.20)	1.04 (0.87–1.24)
Liver cirrhosis								
No	3531	956,226	3.69	1971	472,287	4.17	1.12 (1.06–1.19) ***	1.11 (1.05–1.18) ***
Yes	466	61,846	7.53	327	40,196	8.13	1.08 (0.93–1.24)	1.08 (0.93–1.24)
Osteoporosis								
No	3688	994,420	3.70	2120	500,661	4.23	1.14 (1.08–1.20) ***	1.10 (1.05–1.16) ***
Yes	309	23,652	13.0	178	11,822	15.05	1.15 (0.95–1.38)	1.14 (0.94–1.37)
ESRD								
No	3997	1,018,072	3.92	2298	512,483	4.48	1.14 (1.08–1.20) ***	1.11 (1.05–1.17) ***
Yes	0	0	0	0	0	0	--	--

COPD: chronic obstructive pulmonary disease; ESRD: end-stage renal disease; IHD: ischemic heart disease; PYs: person-years; IR: incidence rate per 1000 PYs; HR: hazard ratio; CI: confidence interval; * *p* < 0.05, ** *p* < 0.01, *** *p* < 0.001; ^#^ Adjusted for age, gender, and the comorbidities enlisted in this table.

**Table 4:** The subgroup analysis risk of hip fractures among the COM patients.

	Hip Fracture	PY	IR	Crude HR	^#^ Adjusted HR
	*n* = 2298			(95%CI)	(95%CI)
Gender					
Female	1195	259,815	4.59	1 (reference)	1 (reference)
Male	1103	252,668	4.36	0.94 (0.87–1.03)	1.19 (1.05–1.34) **
Age					
<20	759	287,443	2.64	1 (reference)	1 (reference)
20–44	438	111,392	3.93	1.53 (1.36–1.72) ***	1.45 (1.22–1.71) ***
45–64	608	81,777	7.43	2.91 (2.61–3.24) ***	1.83 (1.44–2.33) ***
≥65	493	31,871	15.46	6.19 (5.52–6.94) ***	2.85 (2.13–3.80) ***
Outpatient frequencies					
≤3 (times)	1157	270,863	4.27	1 (reference)	1 (reference)
>3	1141	241,620	4.72	1.10 (1.01–1.19) *	0.93 (0.82–1.04)
Hospitalization frequencies					
<1(times)	1953	449,444	4.34	1 (reference)	1 (reference)
≥1	345	63,039	5.47	1.25 (1.11–1.40) ***	1.12 (0.94–1.33)
Hearing loss					
No	1885	468,142	4.02	1 (reference)	1 (reference)
Yes	413	44,341	9.31	2.32 (2.08–2.58) ***	1.21 (1.20–1.42) *
Vertigo					
No	1832	456,977	4.00	1 (reference)	1 (reference)
Yes	466	55,506	8.39	2.10 (1.90–2.33) ***	1.00 (0.85–1.18)
Tinnitus					
No	1851	459,547	4.02	1 (reference)	1 (reference)
Yes	447	52,936	8.44	2.11 (1.90–2.34) ***	1.02 (0.86–1.20)

PY: person-years; IR: incidence rate per 1000 PYs; HR: hazard ratio; CI: confidence interval; * *p* < 0.05, ** *p* < 0.01, *** *p* < 0.001. ^#^ Adjusted for gender, age, outpatient and hospitalization frequencies, hearing loss, vertigo, and tinnitus.

## Data Availability

The data presented in this study are available on request from the corresponding author.
